# Analysis of risk factors for serous exudation of biodegradable material calcium sulfate in the treatment of fracture-related infections

**DOI:** 10.3389/fbioe.2023.1189085

**Published:** 2023-06-06

**Authors:** Bing Du, Yu Su, Dongchen Li, Shuai Ji, Yao Lu, Yibo Xu, Yanling Yang, Kun Zhang, Zhong Li, Teng Ma

**Affiliations:** ^1^ Honghui Hospital, Xi’an Jiaotong University, Xi’an, Shaanxi, China; ^2^ Medical College of Yan’an University, Yan’an, Shaanxi, China

**Keywords:** degradable, fracture-related infection, non-infectious exudation, calcium sulfate, analysis of risk factors

## Abstract

**Objective:** To explore the related risk factors of serous exudation after antibiotic-loaded calcium sulfate treatment of fracture-related infections and to provide a theoretical basis for clinical treatment and prevention of serous exudation complications.

**Methods:** The clinical data of 145 patients with limb fracture-related infection treated with antibiotic-loaded calcium sulfate in Xi’an Honghui Hospital from January 2019 to December 2022 were retrospectively analyzed. All patients were diagnosed with fracture-related infection by preoperative magnetic resonance examination, bacterial culture and gene detection and received antibiotic-loaded calcium sulfate implantation. The postoperative serous exudation was recorded through hospitalization observation, outpatient review or follow-up. The collected clinical data were sorted out, and the patient data were divided into serous exudation groups and non-exudation groups. Firstly, the clinical data of the two groups were compared by single-factor analysis to screen out the risk factors. Then multivariate binary Logistic regression analysis determined the independent risk factors and protective factors.

**Results:** 1) According to the inclusion and exclusion criteria, there were 145 cases with complete clinical data, including 27 cases in the non-infectious exudation group and 118 cases in the non-exudative group; 2) Univariate analysis showed that the history of diabetes, smoking history, calcium sulfate implantation, drainage time, combined flap surgery, geometric shape of implanted calcium sulfate, and thickness of soft tissue covered by the surgical area were all associated with the occurrence of non-infectious exudation after antibiotic-loaded calcium sulfate implantation (*p* < 0.05); 3) The amount of implanted calcium sulfate was more [OR = 5.310, (1.302–21.657), *p* = 0.020], combined with flap surgery [OR = 3.565, (1.195–10.641), *p* = 0.023], and the thickness of soft tissue coverage in the operation area was thinner [OR = 5.305, (1.336–21.057), *p* = 0.018]. Longer drainage time [OR = 0.210, (0.045–0.967), *p* = 0.045] was a protective factor for non-infectious exudation after antibiotic-loaded calcium sulfate implantation.

**Conclusion:** 1) The probability of serous exudation in patients with fracture-associated infection after antibiotic-loaded calcium sulfate surgery was 18.62%. This complication may cause a heavier economic and psychological burden on patients; 2) With the increase of bone infection area and the application of more calcium sulfate, the incidence of serous exudation after antibiotic-loaded calcium sulfate surgery in patients with the fracture-related infection will increase, so we should use the amount of calcium sulfate reasonably on the premise of sufficient control of infection in clinical work, and the incidence of serous exudation will also increase due to the recent skin flap surgery and the thinner soft tissue coverage of calcium sulfate implantation area; 3) Under the premise of being able to drain the drainage from the surgical area, the longer drainage time of the drainage tube has a positive effect on preventing the occurrence of serous exudation.

## Background

Fracture-related infection (FRI) is a common and severe complication of traumatic orthopaedics (Metsemakers et al., 2018). The probability of infection after internal fixation of closed fractures is 1%. The probability of such complications for severe open fractures is as high as 15%–55% (Morgenstern et al., 2018). The diagnosis of FRI is relatively tricky, and it needs to be combined with the medical history, signs, imaging examinations, and bacterial culture to determine comprehensively. For the treatment of FRI, individual differences are significant. The causes of trauma, the site of infection, the duration of infection, and the virulence of pathogenic bacteria are not the same. There is no universal standard for treatment, so there is controversy about treating fracture-related infections (Mauffrey et al., 2019; Simpson and Tsang, 2017). Tibia is the most vulnerable site, accounting for up to 64% (Bezstarosti et al., 2019). Kostas G et al. (Makridis et al., 2013) described the three stages of internal fixation for the treatment of postoperative infection of tibial fractures: Stage I: honeycomb tissue inflammation 2–6 weeks after surgery; stage II: delayed wound healing, exudation, osteonecrosis, pathological fracture; stage III: definite osteomyelitis 9 months or more after surgery. For the treatment of stage I infection, most surgeons prefer conservative treatment (Makridis et al., 2013; Mauffrey et al., 2019; Simpson and Tsang, 2017), while for patients with stage II and III infection, there is no unified treatment plan (Makridis et al., 2013; Simpson and Tsang, 2017). Clinical work found that stage I and stage II infections are relatively hidden. When stage II infection complicated with nonunion occurs, it is usually tricky to distinguish simple nonunion from nonunion complicated with infection. Therefore, many patients have developed a more severe stage III infection, osteomyelitis. Due to the prolonged treatment cycle and high recurrence rate of post-traumatic fracture-related infection and osteomyelitis (Yalikun et al., 2021), single-stage surgery is difficult to cure FRI, especially for more complex infections. Therefore, staged surgery is the current surgical strategy with a higher cure rate. One-stage surgery aims to control infection, and subsequent surgery is for fracture healing (Rice et al., 2021). Microorganisms usually cause this infection. Microorganisms may be introduced during the implantation of internal plants due to incomplete retention of open wound debridement, or they may be brought to the surface of internal plant materials through bacteremia during the perioperative period of fracture. They adhere to and grow on the surface of internal plants to form biofilms. This growth pattern of biofilms improves the resistance of bacteria to the host’s immune system and the use of antibiotics. Biofilms have a protective effect on the growth of bacteria (Gristina and Costerton, 1985). The concentration of antibiotics required to destroy biofilms is hundreds of times that of regular effective antibiotics. It is far beyond ordinary people’s tolerance range (Xu et al., 2022). Therefore, infection control after debridement mainly depends on using local antibiotics (Implanting carrier materials containing antibiotics so that the infected site can directly transport high concentrations of antibiotics). Although polymethylmethacrylate (PMMA) bone cement has been used as a carrier to release this antibiotic for a long time (Wahlig et al., 1984; Buchholz and Engelbrecht, 1970), it has some shortcomings. Its non-absorbable properties need to be removed by secondary surgery. Although it is an effective carrier for antibiotic release, this PMMA particle or mass can be used as a biological material surface carrier for preferential adhesion and growth of microorganisms. It may produce antibiotic resistance (Neut et al., 2001). Several *in vitro* studies on PMMA have shown that although antibiotic-loaded PMMA beads release antibiotics, bacteria can still adhere and grow on antibiotic-loaded bone cement (Kendall et al., 1996). The non-degradability of PMMA usually requires secondary removal and may cause bacterial resistance, which limits its clinical application to some extent. A biodegradable alternative to PMMA is calcium sulfate, which has been reported as an effective tool for the local application of antibiotics (KOVACEVIC, 1953; Fischer and Seidler, 1971; Mackey et al., 1982). Calcium sulfate has good biocompatibility and degradability, avoiding the need for subsequent surgical removal. Studies have shown that it may be an effective material in preventing the formation of bacterial biofilms (Howlin et al., 2016; Howlin et al., 2015). Calcium sulfate also has the advantage of providing bone conduction scaffolds, supporting the growth of new bone when implanted into bone defects (Parker et al., 2011; Walsh et al., 2003). When implanted into soft tissue sites, calcium sulfate can be absorbed entirely and will not cause heterotopic ossification. Compared with PMMA, the additional bactericidal effect of calcium sulfate is more prominent. For some patients with minor bone defects, it can even absorb entirely calcium sulfate to form new bone tissue, avoiding the removal of PMMA in the second stage of operation. With the in-depth study of calcium sulfate, it has been reported that when calcium sulfate is implanted to treat bone infection, the high-concentration calcium sulfate slurry formed during calcium sulfate absorption will flow out of the wound. When the amount of calcium sulfate implanted is large (greater than 20 mL), the incidence of this complication is higher (Beuerlein and McKee, 2010). Gemma (Humm et al., 2014), 21 patients with a bone infection in the study were treated with antibiotic-loaded calcium sulfate, and 7 had serous wound exudation. After the calcium sulfate was absorbed, the wound healed itself, and there was no recurrence of infection. Aditya et al. (Menon et al., 2018) observed the changes in wounds in 39 patients with osteomyelitis after the implantation of calcium sulfate. The results showed that 8 people had wound secretions about 6 days after the operation, of which 2 were purulent exudation, and 6 were serous exudation. The non-infectious wound secretions that appear after the implantation of antibiotic-loaded calcium sulfate can usually subside spontaneously in about 8 weeks. However, it should be noted that it should be distinguished from infectious exudation, mainly based on the results of bacterial culture of exudates, whether there are inflammation in the local incision, and blood test results. The presence of wound secretions alone does not necessarily mean the failure of infection treatment. Although non-infected serous exudation can heal itself, the emergence of this kind of exudation will inevitably interfere with the doctor’s evaluation of the effect of infection treatment. There is even the possibility of misdiagnosis as infectious exudation and secondary surgery. At the same time, even if the diagnosis is correct, a longer wound healing time will undoubtedly prolong the patient’s hospitalization and increase the patient’s physical and mental pressure during treatment. This study used a retrospective study method to review the clinical data of 145 patients who met the inclusion and exclusion criteria and used binary logistic regression analysis to determine independent risk factors and protective factors. In order to guide the clinical to reduce the occurrence of such complications.

## Materials and methods

### Inclusion and exclusion criteria

From January 2019 to December 2022, 145 patients with fracture-related infections who underwent antibiotic-loaded calcium sulfate implantation were included in this study. According to whether the patients had serous exudation within 4 weeks after the operation, the patients were divided into the serous exudation group and the non-exudation group. The hospital ethics committee approved this experiment, and all patients signed the informed consent. The inclusion criteria were: 1) Patients with limb bone infection and osteomyelitis were diagnosed by medical history, clinical manifestations, laboratory examination and imaging examination. 2) Age ≥18 years old; 3) Patients who could tolerate anaesthesia and received antibiotic-loaded calcium sulfate surgery; 4) Patients informed consent and complete clinical data. The exclusion criteria were as follows: 1) Age less than 18 years old; 2) Patients with severe medical diseases who cannot tolerate anaesthesia and surgery; 3) Patients with incomplete clinical data; 4) Patients allergic to calcium sulfate; 5) According to clinical manifestations, bacterial culture, laboratory tests, etc.,.diagnosed as infectious exudation.

### Surgical procedure

**1) Debridement:** 1) Incise the skin, subcutaneous tissue, deep fascia and muscle tissue of the infected site, and remove the infected sinus; the internal fixation material was exposed and removed. 2) The infected soft tissue (Including scar tissue) and pus on the superficial surface of the cortical bone were carefully and gently removed using an orthopaedic spatula or scalpel. 3) According to the range of bone infection determined by preoperative imaging examination, the bone window was enlarged on the cortical bone to facilitate the full exposure of intramedullary infection. The dead bone, pus in the bone marrow cavity, inflammatory granulation, and scar tissue was removed. Then the wound was manually rinsed with more than 1,000 mL of normal saline to expose the sclerotic bone in the bone infection area. The sclerotic bone was removed using a high-speed grinding drill, and the signs of punctate bleeding in the bone tissue were taken as the standard; the “chilli sign” showing good blood supply appeared. 4) Open the bone infection area’s proximal and distal medullary cavity. 5) Use over 3,000 mL of normal saline to wash the wound through the high-voltage pulse system. 6) The saline and bleeding in the bone cavity after debridement were wiped dry.

**FIGURE 1:**
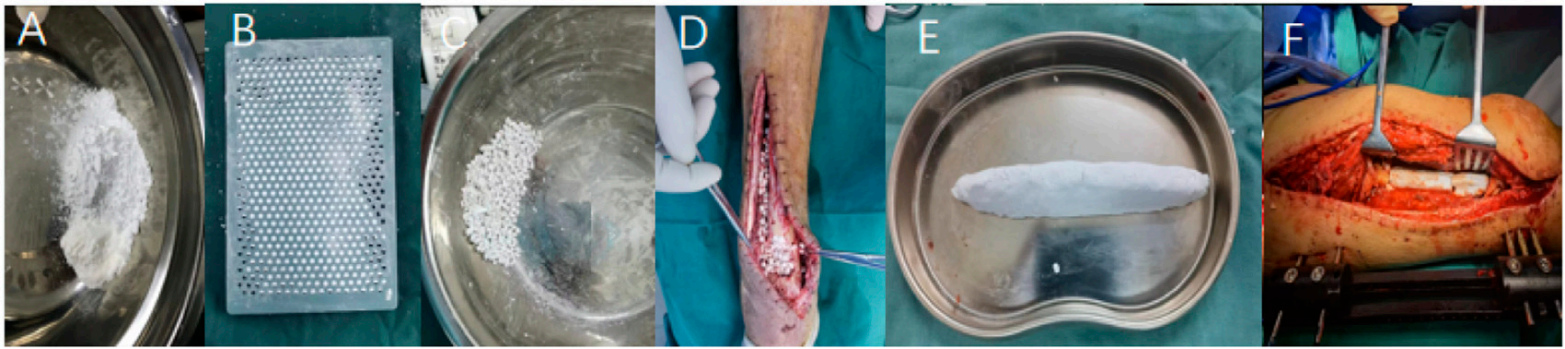
Preparation and implantation effect of calcium sulfate in surgery.

**2) Preparation and implantation of antibiotic-loaded calcium sulfate:** 1) The preparation of antibiotic-loaded calcium sulfate particles was carried out according to the following steps: according to the size of the bone defect, the required amount of medical calcium sulfate powder was poured into a sterile mixing bowl; in a sterile mixing bowl, the antibiotic powder is thoroughly mixed with the calcium sulfate powder according to the ratio of different antibiotics to calcium sulfate; pour an appropriate amount of water for injection into a sterile mixing bowl and stir well until a uniform paste is formed (About 1 min); according to the shape of the lesion, it is shaped and filled or evenly smeared with a layer of paste in the mould provided by the manufacturer to ensure that each particle-hole is filled to prepare complete and uniform particles so that the paste stays in the mould for at least 30 min–60 min after curing, and the expansion mould releases the particles. 2) Implantation of antibiotic-loaded calcium sulfate, the antibiotic-loaded calcium sulfate particles were evenly and dispersedly implanted into the bone defect area on the surface of the inner wall of the medullary cavity. When the calcium sulfate particles were implanted, the external force was avoided. The drainage tube was placed before the closure of the wound, and then the wound was closed by a layered suture. 3) Preparation of bulk antibiotic-loaded calcium sulfate: according to the size of the bone defect, the required amount of medical calcium sulfate powder was poured into a sterile mixing bowl; in a sterile mixing bowl, the antibiotic powder was thoroughly mixed with the calcium sulfate powder according to the ratio of different antibiotics to calcium sulfate; pour an appropriate amount of water for injection into a sterile mixing bowl and thoroughly stir until a uniform paste is formed; the calcium sulfate was shaped according to the geometric shape of the bone defect at the bone graft site, and the preparation was completed after curing for 30 min–60 min. The specific preparation and implantation effect are shown in Figure 1.

For patients with low-toxic infection who had negative preoperative bacterial culture but still had bone infection symptoms or patients with unclear pathogenic bacteria caused by other reasons, microbiological and histopathological examinations were performed on the infected tissues in the wound during the debridement process to facilitate the selection of postoperative antibiotics and the establishment of postoperative pathological diagnosis. The tissue specimens included pus, inflammatory soft tissue and necrotic bone tissue. The amount of calcium sulfate and operation time were recorded.

### Observed indexes

The clinical data of the patients included in the study were collected, including age, gender, smoking history, whether they had medical diseases (Including diabetes, hypertension, heart disease.), operation time, amount of calcium sulfate implanted during operation, geometric shape, thickness of soft tissue covered by the operation area, whether they were combined with flap surgery, type of antibiotics mixed, and drainage tube placement time. The exudation of patients after antibiotic-loaded calcium sulfate treatment was understood through outpatient review and telephone inquiry.

### Criteria for serous exudation

Within 4 weeks after implantation of antibiotic-loaded calcium sulfate, through wound observation during hospitalization, telephone inquiry after discharge, and outpatient review, the wound sinus still exists if there is liquid exudation and precise sinus formation. There is liquid exudation on the second day of dressing change; the serous exudation after implantation of calcium sulfate was determined by laboratory examination and local performance of the exudation.

### Identification of serous exudation and infectious exudation

For all patients with exudation, the exudate samples were sent for bacterial culture and pathogenic microbial gene detection, combined with the characteristics of the exudate (Infectious exudate often shows yellow-white purulent with bloody exudate, while the non-infectious exudate in this study usually shows white paste with bloody exudate in the early stage and clear yellow exudate in the later stage). There is no infection around the sinus. For patients with sinus and exudate manifestations, they were suspected bacterial culture and pathogenic microbial gene detection of false negative and false positive results through further macro gene detection to identify.

### Statistically treated

The case data included in the study were statistically analyzed by SPSS22.0 statistical software (IBM, United States). The patient’s age, gender, smoking history, medical diseases (Including diabetes, hypertension, heart disease.), operation time, the amount of calcium sulfate implanted during the operation, geometric shape, the thickness of the soft tissue covered by the operation area, whether the skin flap surgery, the type of mixed antibiotics, and the time of drainage tube placement were used as independent variables. The dependent variable for univariate analysis was whether the patient had serous exudation. According to preliminary univariate regression analysis results, variables with statistically significant differences were included in the binary logistic regression model for multivariate analysis to determine independent risk factors (*p* < 0.05 was considered statistically significant). The difference in measurement data was compared by normality test and two independent samples *t*-test. The measurement data were expressed as mean ± standard deviation (‾x ± s). The chi-square test performed the comparison of enumeration data. The difference was considered statistically significant when the difference was *p* < 0.05, and the variables with differences could be included in the binary logistic regression model for analysis. The Hosmer-Lemeshow method was used to test the goodness of fit of the regression model. *p* < 0.05 was considered statistically significant.

## Results

**FIGURE 2:**
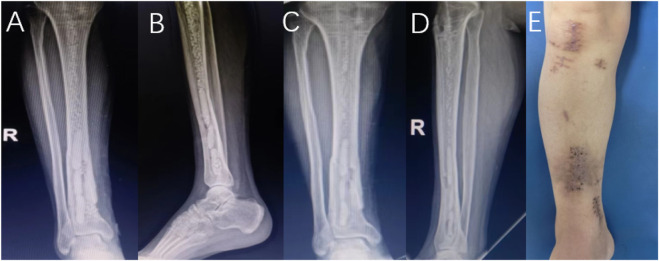
Male patient, 36 years old, no obvious abnormal exudation occurred within 4 weeks after the operation. X-ray **(A,B)** 1 day after antibiotic-loaded calcium sulfate implantation, X-ray **(C,D)** 1 month after the operation, and limb appearance **(E)** 4 weeks after the operation.

**FIGURE 3:**
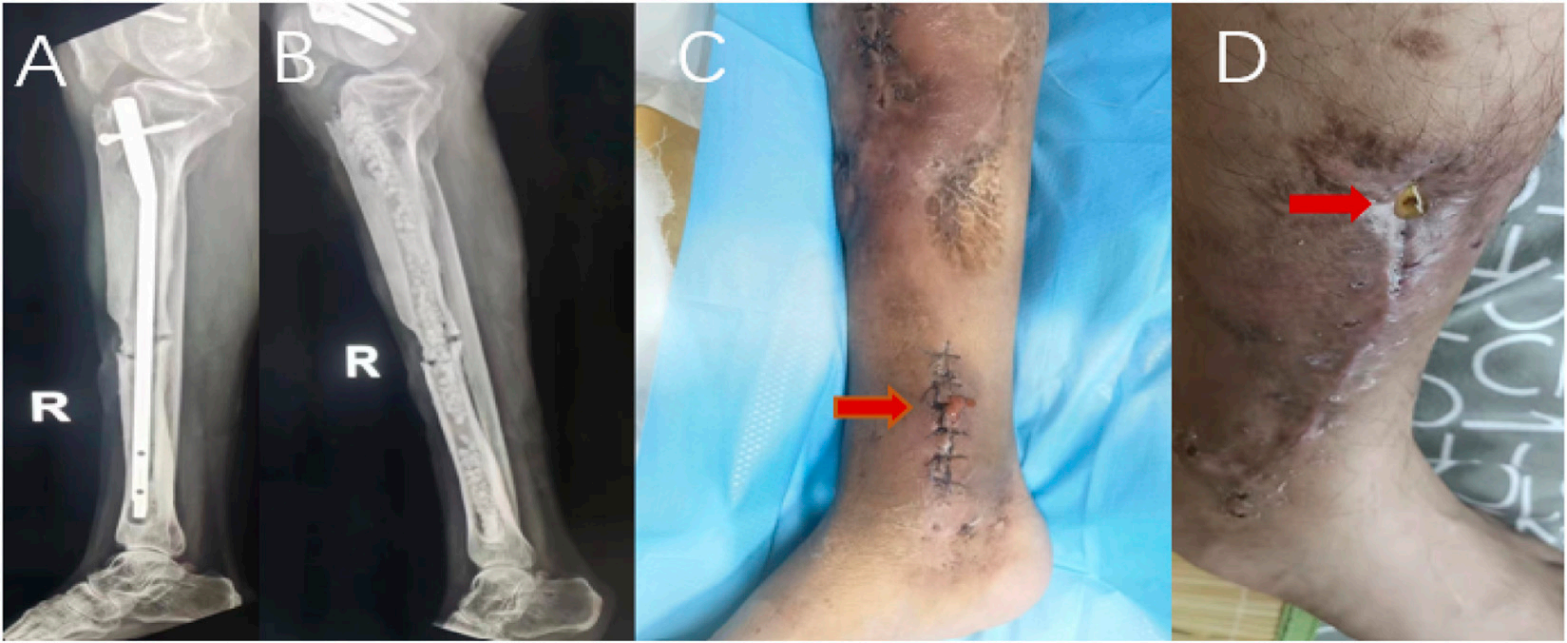
Male patient, 51 years old, X-ray before operation **(A)**, X-ray 1 day after the operation **(B)**, serous exudation appearance of incision on the 6th day after antibiotic-loaded calcium sulfate implantation **(C)**.Another male patient, antibiotic-loaded calcium sulfate implantation 19 days after incision serous exudation appearance **(D)**.

**FIGURE 4:**
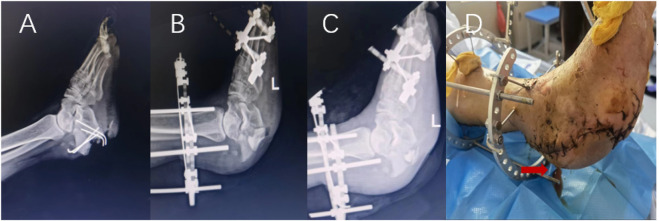
Male patient, 59 years old, X-ray before operation **(A)**, X-ray 1 day after operation **(B)**, X-ray 1 month after operation **(C)**, serous exudation appearance of incision on the 5th day after operation **(D)**.

According to the inclusion and exclusion criteria, this study finally included 145 patients, including 128 males and 17 females; the age ranged from 20 to 84 years, with an average age of 49.6 years. There were 24 patients with diabetes, 18 with hypertension, and 9 with heart disease. Seventy-five patients with a smoking history; the sites of infection involved in the cases included 78 cases of the tibia, 41 cases of the femur, 15 cases of the calcaneus, 5 cases of the patella and 6 cases of the elbow. Calcium sulfate was filled in 93 cases with granular geometry and 52 cases with agglomerate geometry. Calcium sulfate filling surgery combined with local flap surgery in 35 cases; calcium sulfate mixed with antibiotics vancomycin in 44 cases, gentamicin in 39 cases, ceftazidime in 21 cases, imipenem in 10 cases, two or more antibiotics in 31 cases; among the 145 patients, 118 patients did not show abnormal exudation with the degradation of implanted calcium sulfate within 4 weeks. Typical cases are shown in Figure 2. Serous exudation occurred in 27 patients during the degradation of implanted calcium sulfate. Typical cases are shown in Figures 3, 4. Among the 27 patients with serous exudation, 2 patients had eczema at the exudation site. The amount of calcium sulfate implanted was 20.74 ± 7.68 mL in the serous exudation group and 16.14 ± 8.10 mL in the non-exudative group. The drainage days of the serous exudation group was 7.89 ± 1.48 days, and the non-exudative group was 8.83 ± 1.54 days; the operation time was 137.70 ± 30.60 min in the serous exudation group and 135.03 ± 26.19 min in the non-exudative group.

### Serous exudation after antibiotic-loaded calcium sulfate implantation

One hundred forty-eight patients with fracture-related infections received antibiotic-loaded calcium sulfate implantation surgery. According to hospitalization observation records and outpatient review results, 118 patients had good incision healing within 4 weeks after surgery, and no apparent abnormal exudation was observed. Thirty patients had local fluid exudation and sinus formation in the affected limb within 4 weeks after surgery. The exudate samples were sent for bacterial culture and pathogenic microbial gene detection, combined with the exudate characteristics, redness, swelling and pain around the sinus. Among them, 25 patients with local sinus exudation of white paste, pale yellow clear liquid, no obvious redness, swelling and pain, bacterial culture and drug resistance gene detection were negative, considered as serous exudation, included in the study; the local skin temperature of sinus tract formation in 2 patients was high, accompanied by yellow-white purulent exudation. Bacterial culture and drug resistance gene detection were positive, considered infectious exudation and were not included in this study. The local manifestations of the sinus tract and the characteristics of the exudate were considered serous exudation, but the bacterial culture results were all *Staphylococcus aureus*. The pathogenic microbial gene test was positive in 2 cases and negative in 1. In order to exclude the possibility of false positives, further meta-gene detection was performed. The results showed that 2 cases were negative and 1 case was positive. The case was considered a serous exudation and included in the study. One case was infectious exudation and was not included in the study.

In summary, 30 patients with exudation were excluded. Three cases of infectious exudation and 27 cases of serous exudation were excluded. Therefore, 145 cases of antibiotic-loaded calcium sulfate implantation were included in the study. A total of 27 cases of serous exudation occurred, with an incidence of 18.62%.

### Single-factor analysis of serous exudation after antibiotic-loaded calcium sulfate implantation

The results of univariate analysis of the two groups of patients are as follows: Table 1 shows: There was no significant difference in gender (*p* = 0.913), age (*p* = 0.584), hypertension (*p* = 0.820), heart disease (*p* = 0.774), operation time (*p* = 0.644), and mixed antibiotics (*p* = 1.000) in the serous exudation group (*p* > 0.05). However, compared with the non-exudative group, the patients in the non-infectious exudative group had more diabetes (*p* = 0.043), more smoking history (*p* = 0.032), more calcium sulfate implantation (*p* = 0.008), shorter drainage time (*p* = 0.004), more skin flap surgery (<0.001), a higher proportion of granular geometry of intraoperative calcium sulfate implantation (*p* = 0.011), and thinner thickness of soft tissue coverage (*p* < 0.001). The differences were statistically significant (*p* < 0.05).

**TABLE 1:** Single factor analysis of serous exudation after antibiotic-loaded calcium sulfate implantation.

Factor	Slurry exudation group (*n* = 27)	No exudation group (*n* = 118)	χ^2^/t value	*p*-value
Gender (case)				
Male	24	104	0.12	0.913
Female	3	14		
Age (x ± s, year)	51.04 ± 16.00	49.18 ± 15.77	0.549	0.584
Internal diseases(case)				
Diabetes	8	16	4.108	0.043
Hypertension	3	15	0.052	0.820
Heart disease	2	7	0.082	0.774
Smoking history(case)	19	56	4.620	0.032
Calcium sulfate implantation volume(ml)	20.74 ± 7.68	16.14 ± 8.10	2.685	0.008
Drainage time(day)	7.89 ± 1.48	8.83 ± 1.54	−2.891	0.004
Combined flap surgery (case)	15	20	17.884	<0.001
Calcium sulfate form(case)				
Granular (example)	23	70	6.390	0.011
lump (example)	4	48		
Soft tissue of Operation area (case)				
Thickness<1 cm(case)	24	61	12.531	<0.001
Thicknes≥1 cm(case)	3	57		
Operation time(min)	137.70 ± 30.60	135.03 ± 26.19	0.463	0.644
Types of antibiotics (case)				
Vancomycin	8	36	0.043	1.000
Gentamycin	7	32		
Ceftazidime	4	17		
Imipenem	2	8		
Many antibiotics	6	25		

### Multivariate analysis of factors affecting serous exudation after antibiotic-loaded calcium sulfate implantation

The meaningful factors obtained by single factor analysis: history of diabetes, smoking history, amount of calcium sulfate implantation, drainage time, combined flap surgery, geometric shape of calcium sulfate implantation, and thickness of soft tissue covered by the operation area were assigned as independent variables, as shown in Table 2. Binary logistic regression analysis was included. Hosmer-Lemeshow goodness of fit test χ2 = 3.120, *p* = 0.927, indicating that the regression model fits well. Logistic regression analysis showed that the amount of implanted calcium sulfate [OR = 5.310, (1.302–21.657), *p* = 0.020], combined flap surgery [OR = 3.565, (1.195–10.641), *p* = 0.023], and thinner soft tissue coverage [OR = 5.305, (1.336–21.057), *p* = 0.018] were independent risk factors for serous exudation after antibiotic-loaded calcium sulfate implantation. Long drainage time [OR = 0.210, (0.045–0.967), *p* = 0.045] was a protective factor for serous exudation after antibiotic-loaded calcium sulfate implantation, as shown in Table 3.

**TABLE 2:** Each factor assignment description.

Factor	Variable	Description of valuation
Diabetes	X1	No = 0; Yes = 1
Smoking history	X2	No = 0; Yes = 1
Calcium sulfate implantation volume	X3	≤10 = 0; 10–20 = 1; >20 = 2
Drainage time	X4	6∼7d = 0; 8∼9d = 1; ≥10d = 2
Combined flap surgery	X5	No = 0; Yes = 1
Calcium sulfate form	X6	Lump = 0; Granular = 1
Operation area	X7	≥1 cm = 0; <1 cm = 1

**TABLE 3:** Multivariate analysis of serous exudation after antibiotic-loaded calcium sulfate implantation.

Influencing factor	B	SE	Waldχ^2^	OR	95% CI	*p*-value
Calcium sulfate implantation volume	1.670	0.717	5.419	5.310	1.302–21.657	0.020
Drainage time	−1.562	0.780	4.009	0.210	0.045–0.967	0.045
Combined flap surgery	1.271	0.558	5.192	3.565	1.195–10.641	0.023
Operation area	1.669	0.703	5.628	5.305	1.336–21.057	0.018

## Discussion

### Clinical effect of antibiotic-loaded calcium sulfate in the treatment of fracture-related infection

Fracture-related infection is a global health problem. According to statistics, nearly 600,000 artificial joint replacements and 2 million fracture internal fixations in the United States in 2004 eventually led to more than 110,000 infections (Shi et al., 2022). Also, when the site of fracture-related infection involves the foot with diabetes, it can lead to chronic diabetic foot osteomyelitis, increasing the risk of mortality and amputation (Panagopoulos et al., 2015; Demetriou et al., 2013). A study has shown that antibiotics combined with surgical treatment is an effective method for treating bone infections, but about 20% of patients with recurrent infection still exist (Conterno and Turchi, 2013). Due to the damage of blood vessels around the infected bone tissue, it is usually difficult to achieve effective local antibiotic concentration by oral or intravenous drip. The limited antibiotic concentration has little lethality to the biofilm formed by bacteria (Cobb et al., 2020; Maier et al., 2013). Therefore, the focus of antibiotics in treating fracture-related infections lies in the study of local administration systems. Local administration can provide continuous effective antibiotic concentration to the infected site (Zhang et al., 2010), and also avoid the potential toxicity of systemic administration. The local administration of antibiotics began with the emergence of sulfonamides in the 1930 s. In the 1970 s, antibiotics increased the concentration of antibiotics in the local infection area with the introduction of bone cement, while calcium sulfate was discovered in 1970. It was not used in clinical practice until nearly 20 years, and good bone infection treatment was achieved in Europe (McKee et al., 2002). Calcium sulfate has good biocompatibility and is usually completely dissolved within 6–12 weeks of implantation (Beuerlein and McKee, 2010).Calcium sulfate has a certain osteogenic effect, which increases local acidity during dissolution and reabsorption. This may lead to the demineralization of adjacent bone and the release of matrix-binding morphogenetic protein that stimulates bone formation (Yu et al., 2009). At the same time, calcium sulfate promotes bone healing by filling bone voids and preventing the inward growth of fibrous tissue. Over time, calcium sulfate is absorbed, and fibrous vascular tissue replaces its position and eventually forms new blood vessels and bone tissue in this area (Borrelli et al., 2003). When infection combined with bone defect occurs, although the gold standard for treating bone defect is autologous bone transplantation, the amount of autologous bone is limited (Chen et al., 2005). Artificial bone graft material for limb reconstruction of large bone defect is an effective surgical measure (Calori et al., 2011). Studies have compared the bone fusion rate of autologous bone transplantation and calcium sulfate as bone graft substitutes, and found that calcium sulfate and autologous bone also achieved good bone graft effect (Chen et al., 2005). This suggests that antibiotic-loaded calcium sulfate may have a potential advantage in treating infectious bone defects that can replace bone grafts while controlling the progression of infection. However, some experiments have shown that calcium sulfate has a short-term cytotoxicity (Calori et al., 2011), which can lead to inflammation. It will spontaneously cause inflammation after implantation, and even the fracture-related infection is controlled but the inflammation of the surrounding soft tissue has not subsided. Due to the different understanding of the properties of calcium sulfate materials and the inconsistency of surgical procedures, the serous exudation caused by calcium sulfate degradation has been paid more and more attention by orthopedic surgeons, which may lead to the need for secondary surgery or the recurrence of infection. There are many empirical conjectures about the occurrence of this serous exudation. Some scholars believe that it is related to the amount of implantation, some believe that it is related to the destruction of local blood supply and poor soft tissue coverage, but they are all empirical conjectures. The research we have done makes up for the blank that there is only conjecture in clinic and lacks theoretical data support.

### Serous exudation after antibiotic-loaded calcium sulfate implantation

The clinical application of antibiotic-loaded calcium sulfate in treating bone infection has achieved good results. However, we can not ignore the occurrence of related complications caused by the degradability of calcium sulfate itself. Among them, postoperative serous exudation complications are not an example, and all complications of calcium sulfate have a certain proportion. A study of 100 cases of chronic osteomyelitis treated with calcium sulfate-loaded antibiotics (Drampalos et al., 2018) found that 11 patients had wound exudate after surgery, of which 6 patients had exudate that appeared white liquefied calcium sulfate residue. The exudate rate was as high as 33% (Humm et al., 2014). A larger sample size study of 195 cases of chronic osteomyelitis treated with calcium sulfate showed that 30 patients had non-infectious exudate after surgery, which lasted for 2 weeks, and the exudate rate reached 15.4%. Ferguson (Ferguson et al., 2014) found that the proportion of serous exudation after antibiotic-loaded calcium sulfate in the treatment of calcaneal osteomyelitis was as high as 11/34, and the average time of exudation reached 31 days. Ten patients were treated with dressing change dressing, and one patient underwent a second operation to remove the residual calcium sulfate. A total of 145 cases of fracture-related infections treated with antibiotic-loaded calcium sulfate included in this study showed that 27 patients had complications of serous exudation, accounting for 18.6%. Bruce (Ziran et al., 2007) applied calcium sulfate-demineralized bone matrix to treat nonunion and limb bone defects, but found up to 51% (21/41) of exudation, of which 13 patients required surgical intervention drainage, and 14 patients had secondary deep tissue infection after exudation. In summary, in applying antibiotic-loaded calcium sulfate to treating bone infection in multiple medical centers, the problem of serous exudation is more or less encountered. For the treatment of this complication, it can be treated by replacing sterile dressings multiple times. Secondary surgery can also be considered for severe exudation to remove residual calcium sulfate. However, whether it is the heavier psychological and economic burden on patients, or the more frequent dressing change workload brought to orthopedic surgeons, it even needs to be solved by secondary surgery. Moreover, improper treatment after serous exudation causes secondary infection of deep tissue. This suggests that this complication should receive more attention.

### Risk factors affecting serous exudation after antibiotic-loaded calcium sulfate implantation

The serous exudation after calcium sulfate filling has different proportions in different research centers, and the differences are large. This may be related to the differences in the surgical techniques of the surgeons, the different surgical sites included in the study cases, the different sample sizes involved in the study, and the differences in the use of calcium sulfate (Including the amount of use and the geometry of implanted calcium sulfate). Some scholars have found that when the amount of calcium sulfate implanted is greater than 20 mL, the probability of this serous exudation complication will increase (Beuerlein and McKee, 2010), which seems similar to the conclusion obtained in this study. Our study found that the amount of calcium sulfate implanted in the non-exudative group is 16.14 ± 8.16 mL, and the amount of calcium sulfate implanted in the serous exudation group is 21.85 ± 8.22 mL.When the amount of calcium sulfate implanted is greater than 20 mL, the logistic regression model shows that compared with the amount of implantation ≤10 mL. The risk of serous exudation complications increased significantly [OR = 5.310, (1.302–21.657), *p* = 0.020]. A study on applying antibiotic-loaded calcium sulfate in calcaneal osteomyelitis showed abnormal high non-infectious exudative complications (Jiang et al., 2020). The author believes that due to poor soft tissue coverage and physiological lack of blood supply to the calcaneus, the non-infectious exudative complications of the calcaneus are abnormally increased. In this study, it was found that local combined flap surgery [OR = 3.565, (1.195–10.641), *p* = 0.023] and thinner soft tissue coverage [OR = 5.305, (1.336–21.057), *p* = 0.018] were risk factors for serous exudation complications, which reasonably explained why this part of the calcaneus showed a higher incidence of serous exudation complications when calcium sulfate was used to treat bone infections. In addition, our regression model shows that long-term negative pressure drainage is a protective factor for serous exudation [OR = 0.210, (0.045–0.967), *p* = 0.045].There is no definition of reasonable drainage time after antibiotic-loaded calcium sulfate implantation, but this does not mean that the longer the drainage time of negative pressure drainage is, the better. Because long-term drainage itself may cause secondary infection, the length of negative pressure drainage should be determined according to the actual drainage volume of patients. In addition, the geometric shape of calcium sulfate implantation was analyzed by chi-square test in single factor analysis. We found a difference between granular exudation and agglomerated morphology (*p* = 0.011), which was included in the logistic regression model. It was found that geometric shape was not an independent risk factor for serous exudation (*p* > 0.05). However, in clinical work, through outpatient X-ray review, we found that granular calcium sulfate degraded faster, while agglomerated calcium sulfate degraded slower. Therefore, implantation in granular geometry has a potential risk of serous exudation. This needs to be confirmed by later studies with larger sample size.

### Prevention and treatment of serous exudation after antibiotic-loaded calcium sulfate implantation

The risk factors shown in the statistical results of this study were more calcium sulfate filling, combined flap surgery, and thin soft tissue coverage. Protective factors are negative pressure drainage for a long time. Due to the large difference in the condition of patients with fracture-related infections, different patients may have different sites of infection, and the scope of infection is different. The range of dead space caused by osteonecrosis caused by infection is also different. As a result, there is no standard for the effective amount of infection control, and orthopaedic surgeons often must judge the amount required according to experience. However, our study found that more filling was the biggest risk factor, with an OR value of 5.310. For the determination of the amount of implantation, a basic principle followed by this medical center is to determine the size of the infection range according to the preoperative magnetic resonance examination, estimate the amount of calcium sulfate required, and then determine the amount of calcium sulfate combined with the number of dead bone removal and the severity of infection. Therefore, the later research should focus on the minimum effective dosage of calcium sulfate, and reduce the occurrence of serous exudation complications caused by calcium sulfate degradation without affecting infection control. We believe that the serous exudation above the average level after treating calcaneus infection with antibiotic-loaded calcium sulfate is not accidental. The main reason may be that the local soft tissue coverage of the calcaneus is thinner, and the calcaneus is due to its anatomical reasons. The skin edge is prone to infection and necrosis, promoting local sinus tract formation (Jiang et al., 2020). Therefore, when the local combined flap surgery and soft tissue conditions are poor, we should be alert to the occurrence of this complication. The incision can be closely aligned during the suture and the surgical area is fully drained. The occurrence of serous exudation may lead to the occurrence of local eczema, which should be more vigilant in areas with poor soft tissue conditions.

To treat serous exudation complications, it is usually necessary to replace the sterile dressing for a long time to wait for the sinus healing. Due to the excessive amount of exudation, the dressing is often infiltrated and connected with the complex bacterial environment of the outside world, there is a potential risk of nosocomial infection. At the same time, among the 27 patients in the serous exudation group of this study, 2 patients had eczema on the skin around the sinus orifice, which may be related to the exudation of calcium sulfate solution. Therefore, the frequency of dressing change should be at least once a day to prevent potential nosocomial infection and exudate-related eczema. Orthopaedic surgeons who are first contacted with fracture-related infection treatment do not thoroughly understand the serous exudation complications after antibiotic-loaded calcium sulfate filling. Identifying infectious and serous exudation caused by calcium sulfate is difficult. It is easy for young doctors to mistake the occurrence of serous exudation complications as a recurrence of infection, and the wrong choice of secondary surgical treatment brings unnecessary trauma and economic and psychological burden to patients. Currently, the method of identifying infectious and non-infectious exudate is mainly through exudate bacterial culture. However, it should be noted that when *anaerobic bacteria* are infected, the bacteria exposed to the infectious exudate in the air environment may have died, or the normal bacteria such as *Staphylococcus aureus* on the surface of the skin are mixed with the exudate, resulting in false negative or false positive results. Therefore, we should also pay attention to the combination of observing the patient’s incision’s performance and the exudate’s shape. Usually, the same performance can identify infectious exudate and non-infectious exudate. The method of bacterial culture has a long history and has been widely used in clinical practice, but its disadvantage is that it takes a long time and the sensitivity is not high (Tuttle et al., 2011). Macrogene sequencing does not require bacterial culture, and can quickly identify the microbial composition of wound exudate by sequencing, with high sensitivity (Zou et al., 2020). Considering the high cost, not every patient needs to apply this technology. Only when the nature of the exudate is difficult to judge, we use metagenomic detection to identify it, to quickly and comprehensively understand the microbial composition of the exudate and ensure the correct identification of the nature of the patient’s incision exudate. The best time for infection treatment for patients with infectious exudate is not delayed, and patients with non-infectious exudate are not blindly treated with secondary surgery.

### Limitations of the study

This study has some limitations. For example, all the included cases are from Xi’an Honghui Hospital, a single-center retrospective study. The small sample size makes the study’s results biased; the actual condition of patients with fracture-related infection is complex, and the occurrence of non-infectious exudative complications may be related to many factors. The indicators we included in the study may be only part of the relevant factors, and some related important risk factors may be missed. Relying on telephone follow-up patients to determine whether the patient has serous exudation information, due to the lack of medical knowledge of patients, may ignore some cases of exudation complications. In the later stage, we can cooperate with other regional medical centers to expand the sample size of our hospital and improve the analysis of other related risk factors. The mechanism of serous exudation after antibiotic-loaded calcium sulfate implantation is still unclear, and clinical research is also lacking. Further research is needed to understand complications after calcium sulfate implantation, and ultimately improve the treatment of fracture-related infections.

## Conclusion

The probability of serous exudation in patients with fracture-associated infection after antibiotic-loaded calcium sulfate surgery was 18.62%, and this complication may cause heavier economic and psychological burden to patients; with the increase of bone infection area and the application of more calcium sulfate, the incidence of serous exudation after antibiotic-loaded calcium sulfate surgery in patients with fracture-related infection will increase, so we should reasonably use the amount of calcium sulfate under the premise of sufficient control of infection in clinical work. 3 Under the premise of being able to drain the drainage from the surgical area, the longer drainage time of the drainage tube has a positive effect on preventing the occurrence of serous exudation.

## Data Availability

The original contributions presented in the study are included in the article/Supplementary Material, further inquiries can be directed to the corresponding authors.
